# First report of field-evolved resistance to insecticides in *Spodoptera frugiperda* (Lepidoptera: Noctuidae) from Punjab, Pakistan

**DOI:** 10.1371/journal.pone.0324857

**Published:** 2025-06-10

**Authors:** Hafiz Azhar Ali Khan, Ansar Ali

**Affiliations:** 1 Department of Entomology, University of the Punjab, Lahore, Pakistan; 2 Department of Molecular Bioscience, University of Texas at Austin, Austin, United States of America; Government College University Faisalabad, PAKISTAN

## Abstract

The fall armyworm, *Spodoptera frugiperda*, is one of the major destructive pests of agriculture in Pakistan. The widespread use of insecticides for the management of *S. frugiperda* has resulted in the field-evolved resistance to insecticides in different strains worldwide. However, field-evolved resistance to insecticides has not yet been reported in *S. frugiperda* from Pakistan. Following reports of control failure of *S. frugiperda* in Punjab, Pakistan, a study was planned to investigate resistance to insecticides from different classes in field strains of *S. frugiperda* to confirm whether the resistance was indeed evolving. Here, we explored resistance to spinetoram, emamectin benzoate, indoxacarb, diflubenzuron, methoxyfenozide, chlorpyrifos and cypermethrin in seven field strains and compared them with a laboratory susceptible reference (Lab-SF) strain of *S. frugiperda*. Compared with the Lab-SF strain at the LC_50_ levels, the field strains exhibited 24.8–142.7 (spinetoram), 33.4–91.4 (emamectin benzoate), 30.1–90.6 (indoxacarb), 16.1–38.4 (diflubenzuron), 18.4–51.8 (methoxyfenozide), 37.1–222.9 (chlorpyrifos), and 61.9–540.6 (cypermethrin) fold resistance ratios (RRs). In the presence of detoxification enzyme inhibitors [piperonyl butoxide (PBO) and S,S,S-tributyl phosphorotrithioate (DEF)], the toxicity of all the insecticides, with the exception of spinetoram, was significantly enhanced in the tested field strains of *S. frugiperda*, providing insight into the metabolic mechanism of resistance. Additionally, compared with the Lab-SF strain, the resistant field strains exhibited elevated activities of detoxification enzymes such as glutathione S-transferases (GST), carboxylesterases (CarE) and mixed-function oxidases (MFO). Overall, the findings of the present study provide robust evidence of field-evolved resistance to insecticides in *S. frugiperda*, which needs to be managed to minimize yield losses of different crops caused by this global pest.

## Introduction

*Spodoptera frugiperda* (J.E. Smith) (Lepidoptera: Noctuidae), commonly known as the fall armyworm (FAW), is one of the major insect pests of agriculture worldwide [1]. It has the potential to rapidly spread in different regions owing to its strong flight ability [2] and potential to contaminate certain agricultural commodities [3]. It has been reported from different parts of the world. For instance, *S. frugiperda* has been reported from different areas of the Americas and Canada [4–7], Africa and sub-Saharan Africa [8–10], Australia [11,12], Asia and parts of Oceana [13–15]. As it is polyphagous in nature, *S. frugiperda* has been found feeding on a diverse range of wild and cultivated plants. The plants species belonging to the Asteraceae, Poaceae and Fabaceae families are the most preferred hosts of *S. frugiperda* [4]. However, *S. frugiperda* prefers to infests sorghum, *Sorghum bicolor* (L.), and maize, *Zea mays* L., in invaded regions [16].

The climatic conditions of most parts of South Asia and Southeast Asia are usually suitable throughout the year for the growth and expansion of *S. frugiperda* [17,18]. In Pakistan, the first records of *S. frugiperda* were reported from Punjab Province in 2019 [19] and Sindh Province in 2020 [20]. This pest causes significant economic losses in the yield of different crops such as maize, sorghum and cotton [21].

Currently, the use of insecticides is the mainstay for the control of *S. frugiperda* worldwide. Owing to the losses caused by *S. frugiperda*, farmers usually apply high dosages of insecticides. As a result, *S. frugiperda* has developed resistance to various insecticides, which makes its control difficult [22]. Insecticide resistance and pest resurgence are among the major side effects of insecticidal usage to control insect pests of economic importance [23]. Studies have revealed that *S. frugiperda* has developed resistance to various insecticides belonging to different modes of action [24,25]. Field-evolved resistance to insecticides in *S. frugiperda* has been widely reported across different countries, such as Georgia [26], the USA [27], China [28,29], Puerto Rico [30,31], Australia [11,15,32], Egypt [33], India [34] and Brazil [35,36]. In Pakistan, there are several reports of field-evolved resistance to insecticides in different lepidopteran insect pests [24,37–41], which indicate the probability of insecticide resistance in *S. frugiperda*. However, there are no reports of field-evolved resistance to insecticides in *S. frugiperda* from Pakistan.

Elevated activities of detoxifying enzymes are considered one of the major phenomena behind resistance development to most insecticides, which can be confirmed using synergists along with insecticides in bioassays and/or performing enzyme analyses of resistant strains [42]. For example, synergists such as piperonyl butoxide (PBO) and *S,S,S*-tributyl phosphorotrithioate (DEF) have been used in a number of reports to trace the metabolic mechanism of resistance to insecticides in different insect pests [43–48].

Following reports of control failure of *S. frugiperda* in Punjab, Pakistan, and its high potential to rapidly develop resistance to insecticides, a study was planned to investigate resistance to insecticides from different classes in field strains of *S. frugiperda* to confirm whether the resistance was indeed evolving. In addition, we were interested to see whether the metabolic mechanism of resistance is involved in developing resistance to insecticides.

## Materials and methods

### Ethics statement

No specific permit was required to collect *S. frugiperda* samples from farmers’ fields as these were privately owned and collection was made merely by speaking with the private owners. Since, *S. frugiperda* is not an endangered species; no permission was required from any concerned authority in Punjab, Pakistan.

### *Spodoptera frugiperda* strains

Seven field strains and a laboratory susceptible reference strain were used in the present study. Field strains were collected from Multan (30.1864° N, 71.4886° E), Pattoki (31.0249° N, 73.8479° E), Lahore (31.5204° N, 74.3587° E), Mian Channu (30.4390° N, 72.3552° E), Sialkot (32.4945° N, 74.5229° E), Jhang (31.2781° N, 72.3317° E) and Faisalabad (31.4504° N, 73.1350° E) and were designated MTN-SF, PTI-SF, LHR-SF, MCU-SF, SKT-SF, JHG-SF and FSD-SF, respectively. The selected localities were under multiple cropping systems such as maize, cotton, rice, wheat, sugarcane, millet, vegetables and fodders, which were grown side-by-side depending on the season. A variety of recommended insecticides from different classes were in practice in selected localities to manage insect pests of different crops, including *S. frugiperda* [49,50]. Approximately 500 larvae of each field strain were collected from maize fields from 2023 to start rearing in the laboratory. Each field strain was used for bioassays after one generation was reared in the laboratory. A laboratory-susceptible reference strain, designated as “Lab-SF”, was collected in July 2021 from Lahore and reared in the laboratory for two years without exposure to any insecticides. The susceptibility of this strain was the highest to all insecticides compared to that of field strains (please see the results section), and hence it was used as a reference strain. Larvae of all strains were reared on an artificial diet proposed by Truzi et al. [51] and Kasten et al. [52]. The artificial diet was consisted of wheat germ (120 g), white bean (240 g), Brewer’s yeast (72 g), ascorbic acid (7.3 g), sorbic acid (2.4 g), agar (20 g), methylparahydroxybenzoate (4.4 g), vitamin solution (10 ml), formaldehyde 10% (6 ml) and distilled water (1000 ml). Moths were fed a 10% honey solution via soaked cotton pieces in wooden mesh cages (30 × 30 × 30 cm). The laboratory conditions throughout the experiments were 26 ± 2 °C, 12 L: 12 D h photoperiod and 65 ± 5% relative humidity.

### Chemicals

Seven technical-grade insecticides (>95% purity) were used in the bioassays: spinetoram, emamectin benzoate, indoxacarb, diflubenzuron, methoxyfenozide, chlorpyrifos and cypermethrin. Piperonyl butoxide (PBO), an inhibitor of microsomal oxidases (cytochrome P450 monooxygenases) and esterases, and *S,S,S*-tributyl phosphorotrithioate (DEF), an esterase-specific inhibitor, were used in synergism bioassays. All chemicals were from Chem Service, Inc., West Chester, PA.

### Bioassays

The toxicity of different insecticides and synergistic bioassays were performed following the leaf disc bioassay technique [53] using castor bean (*Ricinus communis* L.) leaves. Seven concentrations of each insecticide, resulting in >0% and <100% mortality, were prepared in acetone. Unsprayed, free from insecticidal exposure, castor bean leaves were washed, air-dried and immersed for 10 s in an insecticide solution of a specific concentration and left to dry at ambient temperature. For the control, the leaf discs were immersed in acetone alone. Treated castor bean leaf discs (68 mm diameter) were placed in Petri plates (70 mm diameter) containing moistened filter paper. Ten second-instar larvae were introduced into each Petri plate, and all the bioassays were repeated eight times. Each repetition of bioassays was performed on separate times by preparing fresh solutions of insecticides against second-instar larvae. The bioassay conditions used during the experiments were 26 ± 2 °C, 12 L: 12 D h photoperiod and 65 ± 5% relative humidity. In the case of synergism bioassays, 10 mg/ml PBO or DEF was added to each concentration of insecticide. The concentration of PBO and DEF was the maximum sublethal concentration as determined in preliminary experiments. The synergism bioassays were only conducted with the Lab-SF strain and the most resistant field strain against each insecticide. The mortality of the larvae introduced into each Petri plate was determined after 96 h of exposure. Treated larvae that did not move upon contact with a camel-hair brush were considered dead.

### Enzyme analyses

The protocols described by Caballero et al. [54] and Azhar et al. [55] were followed to assess the activities of detoxification enzymes in the larvae of Lab-SF and field strains. For this purpose, second instar larvae (N = 05) of Lab-SF and each of the field strains were homogenized separately in 400 μL of NaCl (0.15 M) with a chilled mortar in 2 mL Eppendorf tubes for one minute. To obtain the supernatant, the homogenate of each strain was centrifuged at 1397.5 × g for 8 min at 4 °C. These supernatants were used to assess the activities of carboxylesterase (CarE), glutathione-s-transferase (GST) and mixed function oxidase (MFO) following the protocols of Gao et al. [56] and Yang et al. [57], and Bradford [58] method for protein analysis. Briefly, for CarE activity towards α-Na, 10 μL of enzyme solution and 200 μL of substrate solution were added to each well of a 96-well microtiter plate. The optical density was recorded at 450 nm for 10 min, and the standard curve was prepared with α-naphthol [56]. For the activity of GST, CDNB was used as a substrate. The reaction was started by mixing 100 μL each of 1.2 mM CDNB and 6 mM GSH, and 10 μL of the enzyme solution. The optical density was recorded at 340 nm [57]. For the activity of MFO, 100 μL of 2 mM *P*-NA solution and 90 μL enzyme solution were added to each well of microplate and the reaction was started by adding 10 μL of 9.6 mM NADPH. The optical density was recorded at 405 nm for 15 min [56]. All the enzyme analyses were repeated six times.

### Statistical analyses

Mortality data were analyzed by Probit analysis using software PoloPlus (LeOra-Software., 2005) to determine median lethal concentrations (LC_50s_). Any two LC_50_ values were considered significantly different if their 95% CI values did not overlap [59]. The significance of resistance ratios (RRs) was determined following ratio tests by comparing LC_50_ values of field strain with that of the Lab-SF strain. The RR value was significantly different if 95% CI of the ratio did not include one [60].

The data of activities of GST, CarE and MFO in different strains were analyzed by the one-way ANOVA, and means were compared by Tukey’s HSD test using Statistix 8.1v.

## Results

### Bioassay results

The results of the bioassays revealed variable toxicity of the tested insecticides against the Lab-SF and field strains of *S. frugiperda*, which are summarized in Tables 1–3. The recorded LC_50_ value of spinetoram was 0.34 µg/ml for the Lab-SF strain. For field strains, LC_50_ values of spinetoram ranged from 9.97 to 48.52 µg/ml (Table 1). In comparison to the Lab-SF strain, JHG-SF was the most resistant strain to spinetoram (RR = 142.71 fold), followed by the MTN-SF strain (RR = 103.00 fold). The LC_50_ value of emamectin benzoate for the Lab-SF strain was 0.17 µg/ml. The LC_50_ values of emamectin benzoate for field strains ranged from 5.67 to 15.54 µg/ml. Compared with the Lab-SF strain, the JHG-SF strain was the most resistant strain to emamectin benzoate (RR = 91.41 fold) followed by the MCU-SF strain (RR = 72.53 fold). In the case of indoxacarb, the LC_50_ value of the Lab-SF strain was 0.10 µg/ml, while the LC_50_ values of field strains were recorded from 3.01 to 9.06 µg/ml. The PTI-SF strain was the most resistant strain to indoxacarb (RR = 90.60 fold) followed by the SKT-SF, MTN-SF, FSD-SF and JHG-SF strains (RR = 82.40, 78.70, 72.00, and 67.50 fold, respectively) (Table 1).

**Table 1:** Toxicity of spinetoram, emamectin benzoate and indoxacarb against second instar larvae of laboratory and field strains of *S. frugiperda.*

Insecticide	Strain	LC_50_^*^ (95% CI) (µg/ml)	Fit of probit line			
			Slope (SE)	*χ*^2^ (df = 5)	*p*	RR^**^ (95% CI)
Spinetoram	Lab-SF	0.34 (0.26-0.45)	2.30 (0.17)	7.00	0.22	
	MTN-SF	35.02 (29.36-42.47)	1.66 (0.13)	4.12	0.53	103.00 (81.76-130.79)
	PTI-SF	14.61 (10.71-19.93)	1.81 (0.12)	10.29	0.08	42.97 (34.72-53.58)
	LHR-SF	9.97 (7.44-13.11)	1.65 (0.12)	7.32	0.20	29.32 (23.44-36.98)
	MCU-SF	16.06 (13.65-18.90)	1.79 (0.13)	2.77	0.74	47.24 (38.14-58.98)
	SKT-SF	8.43 (6.84-10.21)	1.45 (0.25)	3.67	0.60	24.79 (19.44-31.85)
	JHG-SF	48.52 (40.75-59.06)	1.80 (0.15)	3.88	0.57	142.71 (113.25-181.25)
	FSD-SF	16.88 (13.14-21.77)	2.21 (0.15)	8.87	0.11	49.65 (40.68-61.03)
Emamectin benzoate	Lab-SF	0.17 (0.13-0.21)	2.28 (0.16)	7.52	0.18	
	MTN-SF	9.44 (7.76-11.54)	1.38 (0.11)	1.33	0.93	55.53 (44.78-73.24)
	PTI-SF	6.06 (5.06-7.23)	1.58 (0.24)	3.32	0.65	35.65 (29.19-46.32)
	LHR-SF	7.77 (6.62-9.12)	1.83 (0.13)	3.77	0.58	45.71 (37.99-58.54)
	MCU-SF	12.33 (10.37-14.79)	1.62 (0.26)	2.89	0.72	72.53 (59.47-94.13)
	SKT-SF	5.67 (4.79-6.67)	1.78 (0.15)	0.42	0.99	33.35 (27.59-42.83)
	JHG-SF	15.54 (13.02-18.82)	1.62 (0.30)	1.69	0.89	91.41 (74.58-119.22)
	FSD-SF	7.76 (5.51-10.86)	1.55 (0.12)	8.84	0.12	45.65 (37.70-53.38)
Indoxacarb	Lab-SF	0.10 (0.07-0.13)	1.76 (0.13)	8.86	0.11	
	MTN-SF	7.87 (6.42-9.65)	1.32 (0.10)	2.31	0.80	78.70 (57.83-99.81)
	PTI-SF	9.06 (7.73-10.63)	1.85 (0.13)	2.63	0.76	90.60 (68.68-111.28)
	LHR-SF	3.66 (2.84-4.60)	1.77 (0.13)	5.40	0.37	36.60 (27.46-45.43)
	MCU-SF	3.01 (2.02-4.19)	1.81 (0.22)	10.22	0.07	30.10 (22.57-37.51)
	SKT-SF	8.24 (6.77-10.04)	1.38 (0.11)	4.85	0.43	82.40 (60.86-103.99)
	JHG-SF	6.75 (5.61-8.10)	1.52 (0.28)	1.65	0.90	67.50 (50.36-84.37)
	FSD-SF	7.20 (6.07-8.51)	1.69 (0.12)	4.87	0.43	72.00 (54.22-89.03)

* median lethal concentration (95% confidence interval)

** resistance ratio.

**Table 2:** Toxicity of diflubenzuron and methoxyfenozide against second instar larvae of laboratory and field strains of *S. frugiperda.*

Insecticide	Strain	LC_50_^*^ (95% CI) (µg/ml)	Fit of probit line			
			Slope (SE)	*χ*^2^ (df = 5)	*p*	RR^**^ (95% CI)
Diflubenzuron	Lab-SF	0.13 (0.10-0.16)	2.19 (0.16)	8.51	0.13	
	MTN-SF	2.09 (1.75-2.46)	1.79 (0.13)	3.79	0.58	16.07 (13.23-20.87)
	PTI-SF	2.65 (2.01-3.43)	2.20 (0.15)	9.46	0.09	20.38 (17.07-25.96)
	LHR-SF	3.49 (2.35-5.11)	1.90 (0.27)	10.25	0.07	26.84 (22.34-34.59)
	MCU-SF	4.99 (4.02-6.21)	1.85 (0.13)	5.26	0.38	38.38 (31.81-49.49)
	SKT-SF	2.27 (1.92-2.67)	1.82 (0.12)	4.01	0.55	17.46 (14.44-22.62)
	JHG-SF	2.93 (2.47-3.47)	1.69 (0.30)	4.10	0.54	22.54 (18.57-29.35)
	FSD-SF	3.68 (3.06-4.42)	1.51 (0.11)	2.35	0.80	28.31 (23.06-37.19)
Methoxyfenozide	Lab-SF	0.15 (0.10-0.20)	2.21 (0.15)	10.77	0.06	
	MTN-SF	4.29 (3.62-5.08)	1.69 (0.12)	4.69	0.45	28.60 (23.23-36.50)
	PTI-SF	4.93 (3.82-6.42)	1.94 (0.13)	7.92	0.16	32.87 (27.02-41.57)
	LHR-SF	3.14 (2.57-3.82)	1.39 (0.21)	2.09	0.84	20.93 (16.66-27.38)
	MCU-SF	2.76 (2.15-3.50)	1.81 (0.12)	6.25	0.28	18.40 (15.02-23.41)
	SKT-SF	4.67 (3.97-5.51)	1.77 (0.29)	3.10	0.68	31.13 (25.43-39.66)
	JHG-SF	6.46 (5.53-7.60)	1.90 (0.14)	3.15	0.68	43.07 (37.52-52.10)
	FSD-SF	7.77 (5.94-10.43)	1.85 (0.13)	8.05	0.15	51.80 (42.24-66.00)

* median lethal concentration (95% confidence interval).

** resistance ratio.

**Table 3:** Toxicity of chlorpyrifos and cypermethrin against second instar larvae of laboratory and field strains of *S. frugiperda.*

Insecticide	Strain	LC_50_^*^ (95% CI) (µg/ml)	Fit of probit line			
			Slope (SE)	*χ*^2^ (df = 5)	*p*	RR^**^ (95% CI)
Chlorpyrifos	Lab-SF	0.63 (0.50-0.78)	2.45 (0.18)	4.96	0.42	
	MTN-SF	93.44 (76.05-118.56)	1.45 (0.24)	2.80	0.73	148.32 (114.38-193.19)
	PTI-SF	51.98 (44.36-61.33)	1.87 (0.13)	2.35	0.80	82.51 (66.76-102.14)
	LHR-SF	66.41 (55.42-80.85)	1.59 (0.22)	2.84	0.72	105.41 (83.53-133.64)
	MCU-SF	113.90 (92.11-146.59)	1.49 (0.12)	2.26	0.81	180.79 (138.25-237.50)
	SKT-SF	23.36 (19.34-28.03)	1.52 (0.34)	1.82	0.87	37.08 (29.46-46.90)
	JHG-SF	140.41 (111.27-187.14)	1.44 (0.13)	2.88	0.72	222.87 (166.50-299.69)
	FSD-SF	104.59 (85.86-131.53)	1.58 (0.32)	2.32	0.80	166.02 (128.99-214.65)
Cypermethrin	Lab-SF	0.79 (0.64-0.97)	2.60 (0.19)	4.94	0.42	
	MTN-SF	156.96 (122.67-214.43)	1.40 (0.25)	3.17	0.67	198.68 (146.80-271.82)
	PTI-SF	89.68 (74.22-111.15)	1.60 (0.36)	1.98	0.85	113.52 (89.56-145.46)
	LHR-SF	59.26 (44.50-81.37)	1.72 (0.41)	8.37	0.14	75.01 (60.52-94.00)
	MCU-SF	48.92 (38.36-63.49)	1.54 (0.12)	5.24	0.39	61.92 (49.48-78.34)
	SKT-SF	107.33 (87.56-136.65)	1.53 (0.14)	4.30	0.51	135.86 (105.50-176.85)
	JHG-SF	234.37 (174.64-349.70)	1.38 (0.35)	1.52	0.91	296.67 (206.58-430.66)
	FSD-SF	427.07 (261.28-913.13)	0.97 (0.13)	1.22	0.94	540.59 (292.94-808.30)

* median lethal concentration (95% confidence interval).

** resistance ratio.

For IGRs, the recorded LC_50_ value of diflubenzuron was 0.13 µg/ml for the Lab-SF strain. For the field strains, LC_50_ values of diflubenzuron ranged from 2.09 to 4.99 µg/ml (Table 2). The RR values for different field strains ranged from 16.07–38.38 folds greater than those of the Lab-SF strain. In the case of methoxyfenozide, the LC_50_ value of the Lab-SF strain was 0.15 µg/ml. Among the different field strains, the MCU-SF strain was observed as the most susceptible strain, with the LC_50_ value of 2.76 µg/ml, whereas the FSD-SF was the least susceptible strain (LC_50_ = 7.77 µg/ml). Overall, the RRs were 18.40–51.80 folds greater for different field strains than for the Lab-SF strain.

On the basis of the recorded LC_50_ values, chlorpyrifos and cypermethrin were the least toxic insecticides to the Lab-SF strain and field strains compared with the other insecticides (Table 3). The LC_50_ value of chlorpyrifos was 0.63 µg/ml for the Lab-SF strain. The field strains exhibited LC_50_ values ranging from 23.36 to 140.41 µg/ml for chlorpyrifos. Compared with the Lab-SF strain, the JHG-SF strain was the most resistant strain to chlorpyrifos (RR = 222.87 fold) followed by the MCU-SF (RR = 180.79 fold), FSD-SF (RR = 166.02 fold) and MTN-SF (RR = 148.32 fold) strains (Table 3). The recorded LC_50_ value of cypermethrin was 0.79 µg/ml for the Lab-SF strain. For the field strains, LC_50_ values of cypermethrin ranged from 48.92 to 427.07 µg/ml. In the case of alpha-cypermethrin, the FSD-SF strain was the most resistant strain (RR = 540.59 fold) compared with the Lab-SF strain (Table 3).

### Synergism analyses

In synergism bioassays, based on non-significant synergism ratios, PBO and DEF did not significantly synergize with the toxicity of any insecticide tested against the Lab-SF strain (Table 4). In addition, PBO and DEF also failed to synergize with the toxicity of spinetoram in the field strain (JHG-SF). However, indoxacarb, emamectin benzoate, diflubenzuron, methoxyfenozide, chlorpyrifos, and cypermethrin exhibited significantly enhanced toxicity in the tested field strains of *S. frugiperda* (Table 4).

**Table 4:** Toxicity of insecticides alone and in combination with synergists (PBO or DEF) against second instar larvae of laboratory and field strains of *S. frugiperda.*

Strain	Insecticide	LC_50_^*^ (95% CI) (µg/ml)	Fit of probit line			
			Slope (SE)	*χ*^2^ (df = 5)	*p*	SR^**^ (95% CI)
Lab-SF	Spinetoram alone	0.34 (0.26-0.45)	2.30 (0.17)	7.00	0.22	
	Spinetoram+PBO	0.36 (0.28-0.46)	1.99 (0.34)	4.62	0.46	0.94 (0.77-1.71)
	Spinetoram+DEF	0.30 (0.21-0.42)	1.76 (0.14)	7.53	0.18	1.13 (0.90-1.42)
JHG-SF	Spinetoram alone	48.52 (40.75-59.06)	1.80 (0.15)	3.88	0.57	
	Spinetoram+PBO	53.98 (39.48-83.89)	1.24 (0.21)	1.12	0.95	0.90 (0.60-1.36)
	Spinetoram+DEF	44.17 (34.43-61.28)	1.47 (0.37)	2.61	0.76	1.09 (0.78-1.55)
Lab-SF	Emamectin benzoate alone	0.17 (0.13-0.21)	2.28 (0.16)	7.52	0.18	
	Emamectin benzoate+PBO	0.16 (0.14-0.19)	1.86 (0.14)	0.62	0.99	1.06 (0.81-1.26)
	Emamectin benzoate+DEF	0.18 (0.16-0.21)	2.68 (0.20)	2.98	0.70	0.94 (0.75-1.10)
JHG-SF	Emamectin benzoate alone	15.54 (13.02-18.82)	1.62 (0.30)	1.69	0.89	
	Emamectin benzoate+PBO	7.65 (6.22-9.55)	1.36 (0.13)	0.88	0.97	2.03 (1.53-2.70)
	Emamectin benzoate+DEF	10.17 (8.30-12.70)	1.44 (0.41)	0.19	0.99	1.53 (1.15-2.03)
Lab-SF	Indoxacarb alone	0.10 (0.07-0.13)	1.76 (0.13)	8.86	0.11	
	Indoxacarb+PBO	0.13 (0.11-0.16)	1.63 (0.14)	1.08	0.96	0.77 (0.61-1.02)
	Indoxacarb+DEF	0.11 (0.08-0.15)	1.46 (0.35)	4.90	0.43	0.91 (0.72-1.24)
PTI-SF	Indoxacarb alone	9.06 (7.73-10.63)	1.85 (0.13)	2.63	0.76	
	Indoxacarb+PBO	5.47 (4.68-6.40)	1.97 (0.15)	1.91	0.86	1.66 (1.32-2.07)
	Indoxacarb+DEF	4.44 (3.71-5.27)	1.71 (0.36)	3.65	0.60	2.04 (1.61-2.59)
Lab-SF	Diflubenzuron alone	0.13 (0.10-0.16)	2.19 (0.16)	8.51	0.13	
	Diflubenzuron+PBO	0.14 (0.11-0.17)	2.02 (0.44)	2.72	0.74	0.93 (0.76-1.17)
	Diflubenzuron+DEF	0.15 (0.11-0.21)	2.32 (0.17)	10.25	0.07	0.87 (0.69-1.04)
MCU-SF	Diflubenzuron alone	4.99 (4.02-6.21)	1.85 (0.13)	5.26	0.38	
	Diflubenzuron+PBO	2.35 (1.97-2.78)	1.74 (0.14)	0.55	0.99	2.12 (1.68-2.70)
	Diflubenzuron+DEF	2.47 (1.96-3.09)	2.02 (0.37)	4.18	0.52	2.02 (1.61-2.53)
Lab-SF	Methoxyfenozide alone	0.15 (0.10-0.20)	2.21 (0.15)	10.77	0.06	
	Methoxyfenozide+PBO	0.12 (0.10-0.14)	2.44 (0.18)	1.79	0.88	1.25 (0.85-1.52)
	Methoxyfenozide+DEF	0.12 (0.08-0.16)	2.01 (0.25)	9.13	0.10	1.25 (0.91-1.39)
FSD-SF	Methoxyfenozide alone	7.77 (5.94-10.43)	1.85 (0.13)	8.05	0.15	
	Methoxyfenozide+PBO	4.11 (3.53-4.82)	2.04 (0.16)	2.78	0.73	1.89 (1.50-2.38)
	Methoxyfenozide+DEF	3.69 (3.20-4.26)	2.27 (0.27)	2.96	0.71	2.11 (1.69-2.63)
Lab-SF	Chlorpyrifos alone	0.63 (0.50-0.78)	2.45 (0.18)	4.96	0.42	
	Chlorpyrifos+PBO	0.61 (0.46-0.81)	2.50 (0.34)	8.17	0.15	1.03 (0.85-1.26)
	Chlorpyrifos+DEF	0.59 (0.45-0.79)	2.59 (0.29)	8.63	0.12	1.07 (0.88-1.29)
JHG-SF	Chlorpyrifos alone	140.41 (111.27-187.14)	1.44 (0.13)	2.88	0.72	
	Chlorpyrifos+PBO	30.47 (23.68-39.98)	1.66 (0.52)	4.02	0.55	4.61 (3.35-6.33)
	Chlorpyrifos+DEF	48.84 (39.58-62.49)	1.44 (0.14)	1.64	0.90	2.87 (2.04-4.06)
Lab-SF	Cypermethrin alone	0.79 (0.64-0.97)	2.60 (0.19)	4.94	0.42	
	Cypermethrin+PBO	0.69 (0.56-0.85)	2.28 (0.25)	4.13	0.53	1.14 (0.94-1.40)
	Cypermethrin+DEF	0.83 (0.63-1.14)	1.92 (0.14)	6.56	0.26	0.95 (0.77-1.17)
FSD-SF	Cypermethrin alone	427.07 (261.28-913.13)	0.97 (0.13)	1.22	0.94	
	Cypermethrin+PBO	51.36 (39.67-70.85)	1.15 (0.34)	0.21	0.99	8.32 (4.26-16.22)
	Cypermethrin+DEF	73.84 (55.82-107.15)	1.18 (0.25)	0.78	0.98	5.78 (2.92-11.46)

* median lethal concentration (95% confidence interval).

** synergism ratio calculated as LC_50_ of insecticide alone divided by LC_50_ of insecticide along with PBO or DEF.

### Detoxification enzyme analyses

**Fig 1:**
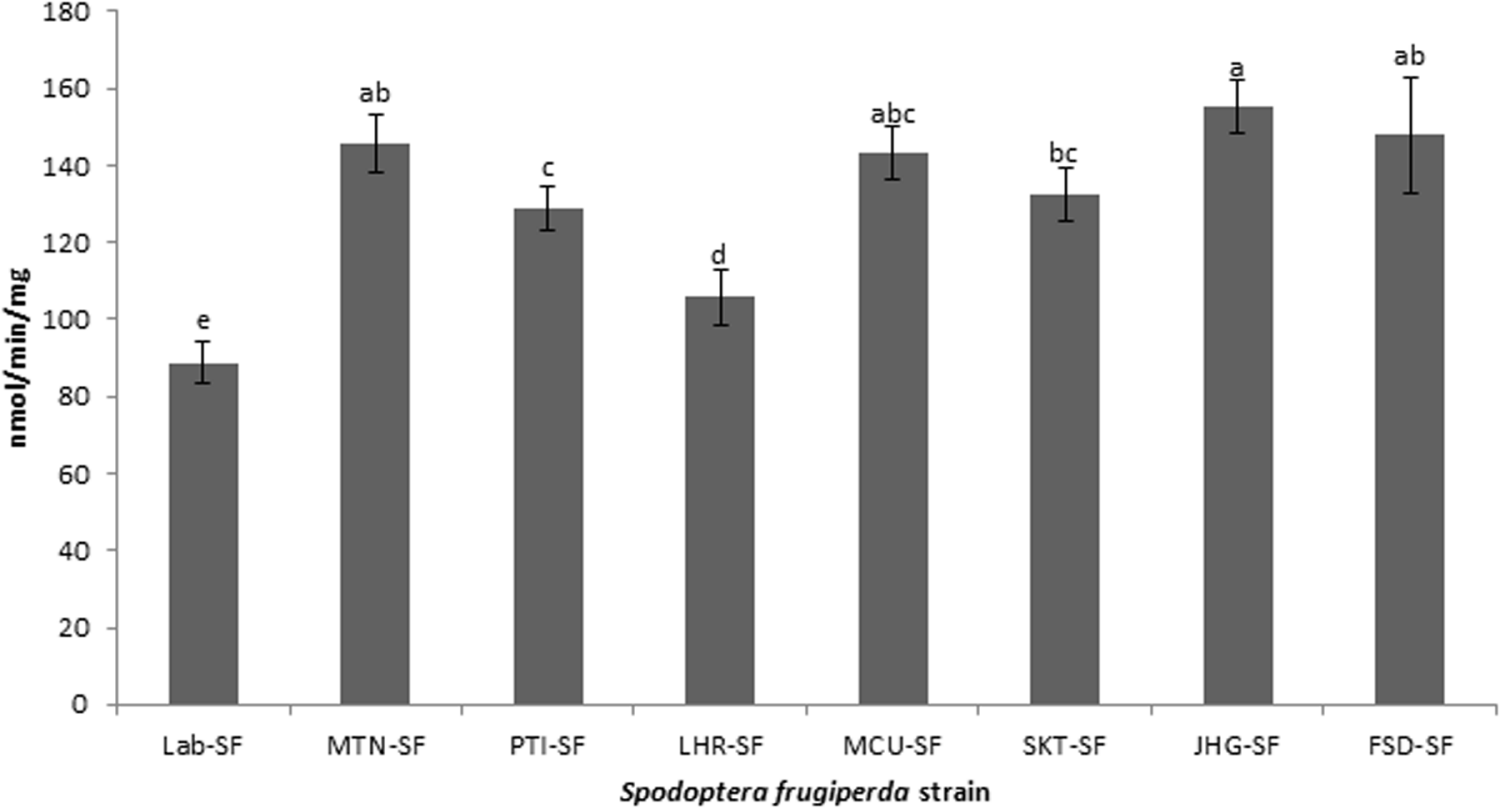
Activities of carboxylesterase (CarE) in laboratory and field strains of *Spodoptera frugiperda* collected from maize fields. Activities of CarE in different strains were analyzed by one-way ANOVA and means were compared with Tukey’s HSD test at p ≤ 0.05. Data bars (mean±SE) with different letters are significantly different (df = 7,32; F = 39.1; p < 0.01).

**Fig 2:**
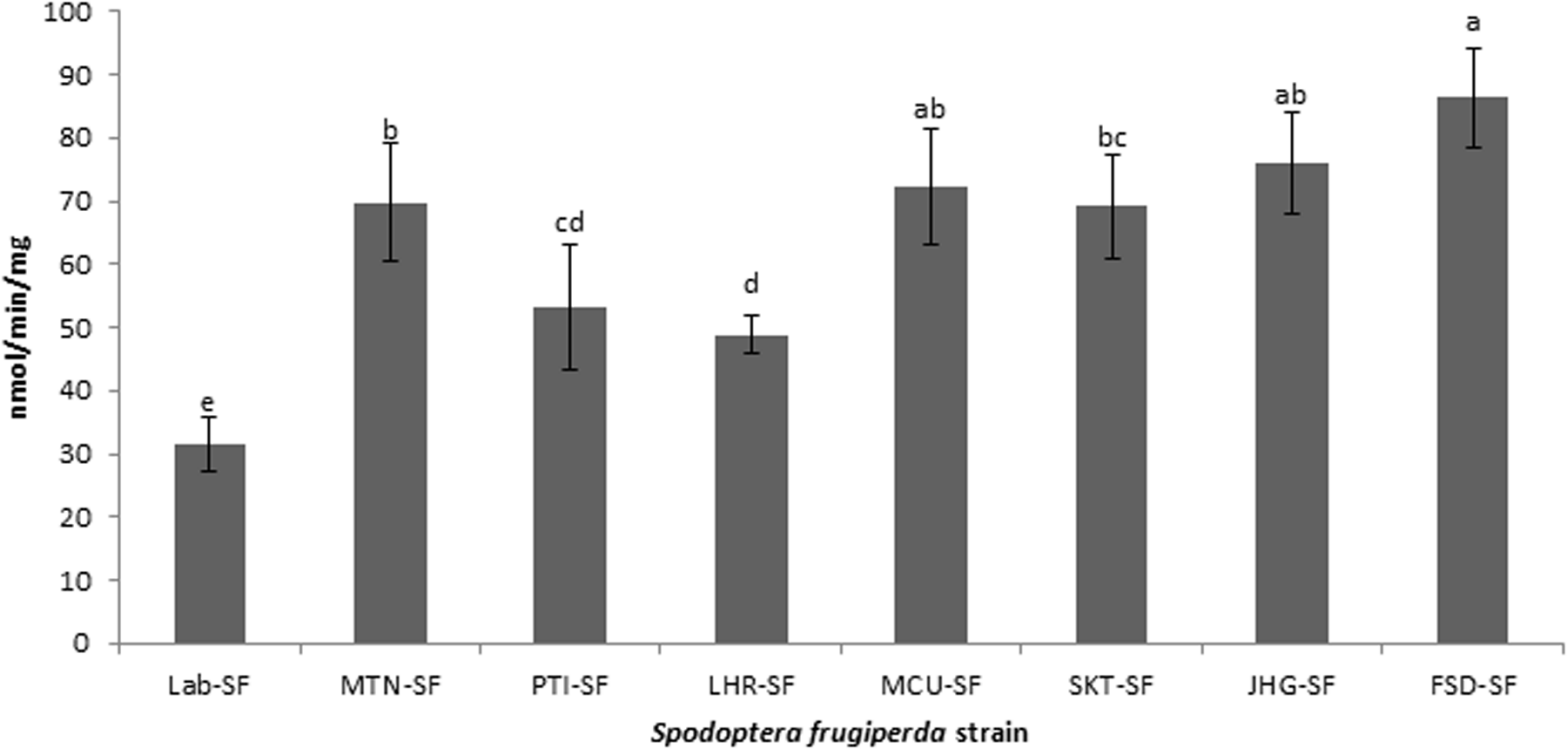
Activities of glutathione-s-transferase (GST) in laboratory and field strains of *Spodoptera frugiperda* collected from maize fields. Activities of GST in different strains were analyzed by one-way ANOVA and means were compared with Tukey’s HSD test at p ≤ 0.05. Data bars (mean±SE) with different letters are significantly different (df = 7,32; F = 25.4; p < 0.01).

**Fig 3:**
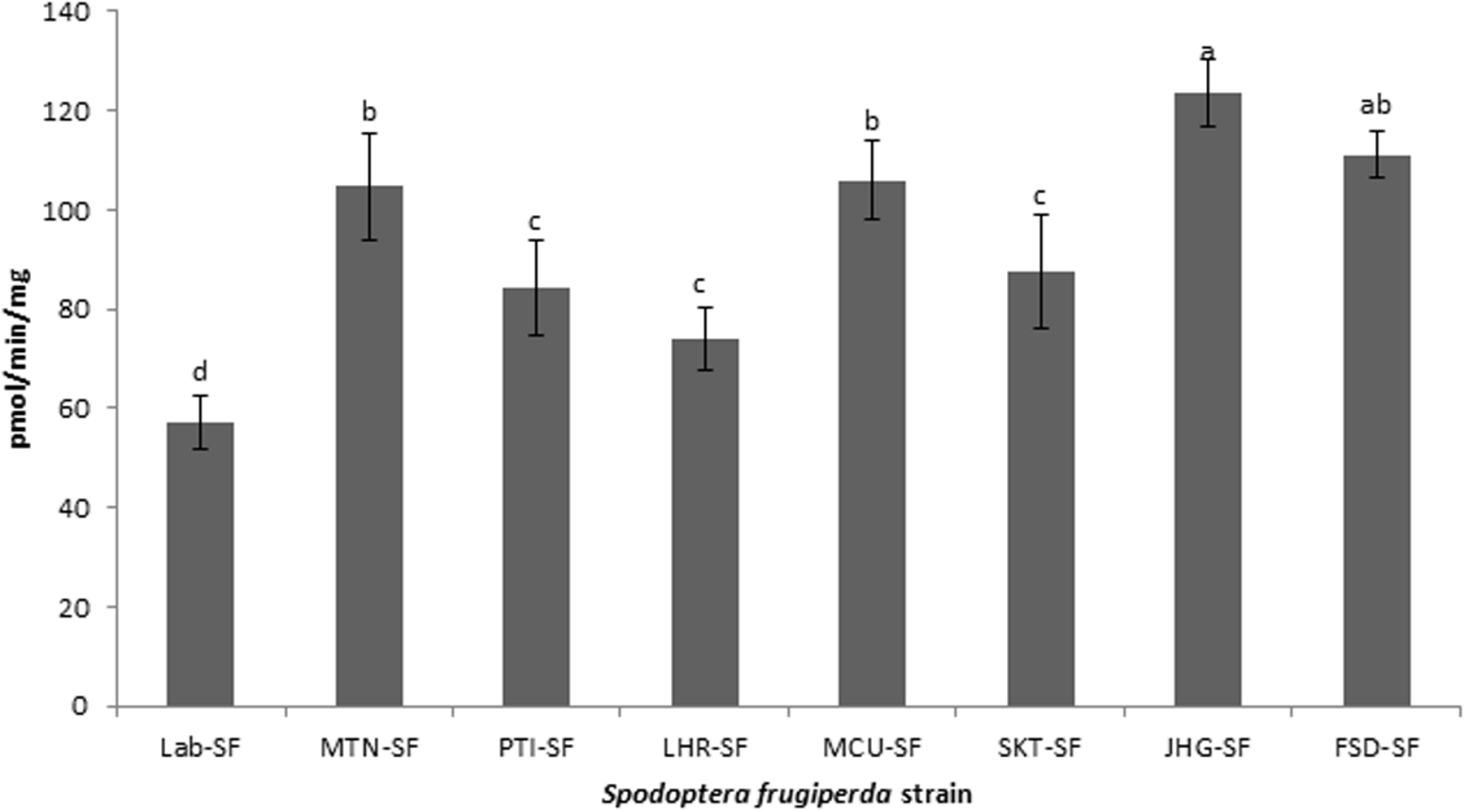
Activities of mixed function oxidase (MFO) in laboratory and field strains of *Spodoptera frugiperda* collected from maize fields. Activities of MFO in different strains were analyzed by one-way ANOVA and means were compared with Tukey’s HSD test at p ≤ 0.05. Data bars (mean±SE) with different letters are significantly different (df = 7,32; F = 35.3; p < 0.01).

All the field strains of *S. frugiperda* exhibited significantly higher activities of CarE, GST and MFO than did the Lab-SF strain (Figs 1-3). The highest activity of CarE (Fig 1) was detected in the JHG-SF strain, followed by the FSD-SF, MTN-SF and MCU-SF strains, whereas the lowest activity was observed in the Lab-SF strain (df = 7,32; F = 39.1; p < 0.01). In the case of GST (Fig 2), the FSD-SF strain exhibited the highest activity, followed by the JHG-SF, MCU-SF, MTN-SF and SKT-SF strains (df = 7,32; F = 25.4; p < 0.01). The JHG-SF strain presented the highest activity of MFO (Fig 3), followed by the FSD-SF, MCU-SF and MTN-SF strains, while SKT-SF, PTI-SF and LHR-SF showed the lowest activity compared with the other field strains (df = 7,32; F = 35.3; p < 0.01). Overall, the lowest activities of CarE, GST and MFO were recorded in the Lab-SF strain (Figs 1-3).

## Discussion

The management of insect pests with the help of synthetic insecticides is an important tool; however, misuse or extensive use of insecticides could lead to the development of resistance in target pests over time [23]. Understanding the current susceptibility levels of a specific insect pest to different insecticides could help strengthen resistance monitoring and management practices. A variety of insecticides from the organophosphate, carbamate, pyrethroid, and growth regulator classes are used to manage *S. frugiperda* worldwide [25,61–66]. This has resulted in the development of resistance to insecticides in *S. frugiperda* in different countries [11,27,29,33,67]. Therefore, it is necessary to investigate patterns of resistance development to diverse insecticides in target pests, such as *S. frugiperda*, in different regions in order to effectively manage resistance problems [47].

The results of the present study revealed notable resistance to insecticides from different classes in Pakistani strains of *S. frugiperda*. In addition, insecticidal bioassays in the presence of synergists and enzyme analyses of field strains indicated the possibility of a metabolic mechanism of resistance to most of the tested insecticides. In India, *S. frugiperda* was recently reported to be resistant to chlorantraniliprole, flubendiamide, chlorpyrifos, thiodicarb and deltamethrin [68]. Moreover, minor to low levels of resistance to indoxacarb, chlorantraniliprole and emamectin benzoate were detected in different field strains of *S. frugiperda* from China [29]. These reports, including the results of the present study, indicate that South Asian and East Asian strains of *S. frugiperda* have the potential to develop resistance to a wide range of insecticides from different classes. Recent reports of insecticidal control failure and subsequent outbreaks in *S. frugiperda* might be associated with the development of resistance to a diverse range of insecticides. As stated earlier, *S. frugiperda* has been reported in Punjab, Pakistan during 2019 [19]. Higher levels of resistance in Pakistani strains of *S. frugiperda* than those reported in Indian [68] and Chinese strains [29] might be due to the reason that *S. frugiperda* migrated into Pakistan from nearby countries already had resistance gene(s). The resistance gene(s) probably helped Pakistani strains of *S. frugiperda* to develop high levels of resistance in a short period after their possible exposures with a variety of insecticides used in Punjab, Pakistan.

Spinetoram is a biological compound obtained from the fermentation process of *Saccharopolyspora spinosa* (a soil actinomycete). In insects, spinetoram disrupts neuronal activity by interfering with GABA-gated ion channels and activating nicotinic acetylcholine receptors (nAChRs) in the nervous system [69]. Resistance to spinosyns (spinosad or spinetoram) in *S. frugiperda* has been reported in Brazil [70,71], Puerto Rico and Mexico [72], and China [73]. In the present study, *S. frugiperda* strains exhibited 29.32 to 103.00 fold resistance to spinetoram in comparison with the laboratory susceptible reference strain, and there was considerable variation in resistance among the tested field strains. In contrast, 16 field strains of *S. frugiperda* from China exhibited 1.40 to 7.65 fold resistance to spinetoram, with very low intraspecific variation in resistance [74]. Similarly, Australian strains of *S. frugiperda* showed no resistance to spinetoram compared with a reference strain of *Helicoverpa armigera* (Hübner) [11]. In Pakistan, spinetoram has been in use against different lepidopteran insect pests since 2011 [49]. The high levels of resistance to spinetoram in the present study might be due to its frequent use against a number of insect pests of different field crops, including *S. frugiperda*. Previously, resistance to spinetoram in other lepidopteran pests has also been reported from Punjab, Pakistan [37–39]. The data of the present study revealed that resistance to emamectin benzoate and indoxacarb in different field strains ranged from 33.35 to 91.41 fold and from 30.10 to 90.60 fold, respectively, than that in the Lab-SF strain. Similar to spinetoram, field strains showed variable intraspecific toxicity to emamectin benzoate and indoxacarb. Previously, resistance to emamectin benzoate has been reported in different strains of *S. frugiperda* [75–77] and closely related species, such as *S. litura* [38,78] and *S. exigua* [79,80]. Similarly, *Spodoptera* species have also expressed resistance to indoxacarb in different areas [53,81–87]. In contrast to the present study, field strains of *S. frugiperda* from Egypt exhibited no resistance to emamectin benzoate [33]. The high levels of resistance to these insecticides in the present study might be due to their frequent use against a number of insect pests of different field crops, including *S. frugiperda*.

Our data revealed that, compared with other strains, most of the field strains of *S. frugiperda* were relatively less resistant to diflubenzuron and methoxyfenozide. Diflubenzuron and methoxyfenozide are insect growth regulators: diflubenzuron is a chitin synthesis inhibitor [88], whereas methoxyfenozide is a molting hormone that causes insect mortality by activating the premature molting process [89]. In contrast to the data of the present study, methoxyfenozide had lower toxicity than spinosyns and emamectin benzoate to different field strains of *S. frugiperda* in Australia [11]. Similarly, Egyptian strains of *S. frugiperda* exhibited no resistance to diflubenzuron [33]. Owing to the highly specific mode of action of diflubenzuron and methoxyfenozide and their low resistance compared with the other insecticides, they are suitable candidates that can be used in rotation in order to manage resistance problems in *S. frugiperda*.

The results of the present study revealed that, compared with the other tested insecticides, chlorpyrifos and cypermethrin were the least toxic insecticides against most of the field strains of *S. frugiperda*. In comparison to the Lab-SF strain, resistance to chlorpyrifos and cypermethrin in field strains ranged from 37.08 to 222.87 fold and 61.92 to 540.59 fold, respectively. Chlorpyrifos and cypermethrin have been widely used for the management of *S. frugiperda*. However, many cases have reported resistance to these insecticides. Resistance to chlorpyrifos has been reported in different strains of *S. frugiperda* from different countries [11,27,28,72,90–92]. Similarly, Australian strains showed 44–132 fold reduced toxicity to cypermethrin in comparison with a reference strain of *H. armigera*.

Metabolic detoxification is one of the major mechanisms behind resistance development to insecticides [93]. The presence of a metabolic mechanism of resistance can initially be checked by conducting insecticidal bioassays along with synergists such as PBO and DEF [23,94]. The data of the present study exhibited that the toxicity of emamectin benzoate, indoxacarb, diflubenzuron, methoxyfenozide, chlorpyrifos and cypermethrin significantly synergized with PBO and DEF in the tested field strains of *S. frugiperda*. These results indicate the involvement of monooxygenases and esterases in imparting resistance to different insecticides in the tested field strains of *S. frugiperda*. Such type of synergism has also been reported in *S. frugiperda* and some other *Spodoptera* spp. For instance, PBO had a significant effect on synergizing toxicity of emamectin benzoate and indoxacarb in *S. frugiperda* [29]. In another report, PBO significantly synergized with the toxicity of indoxacarb in a *S. frugiperda* strain [83]. PBO and DEF have been reported to significantly suppress resistance to pyrethroid and organophosphate, respectively, in Indian strains of *S. litura* [95]. Similarly, DEF significantly enhanced the toxicity of cypermethrin in *S. frugiperda* [96]. Both PBO and DEF failed to enhance the toxicity of spinetoram, which is in broad agreement with our previous reports where PBO and DEF failed to synergize with the toxicity of spinosyn in different insect pests [97–100]. The presence of a metabolic mechanism of resistance was further confirmed by assessing detoxification enzyme activities in *S. frugiperda* strains. Metabolic enzymes such as GSTs, CarE and MFO help detoxify insecticides in insect pests [101,102]. The data of the present study revealed that all the field strains of *S. frugiperda* exhibited significantly higher activities of CarE, GST and MFO than did the Lab-SF strain. Hence, further studies on the metabolic mechanisms of resistance to insecticides in *S. frugiperda* could help in formulating resistance management strategies. For example, proteomics studies in resistant insects can be helpful to identify insect proteins interacting with insecticides, and their modifications can be characterized [103].

### Conclusion

*Spodoptera frugiperda* is a relatively new pest of field crops in Pakistan. The presence of notable levels of resistance to diverse insecticides makes its management difficult. The integration of nonchemical measures along with insecticides, aimed at reducing insecticidal usage, could be helpful for managing resistance problems and effectively managing *S. frugiperda*. The data of the present study showed that *S. frugiperda* is still susceptible to IGRs. Therefore, the rotational use of IGRs with other insecticides could be helpful for managing *S. frugiperda*. Further studies are needed to study the nature of the development of resistance to insecticides in *S. frugiperda* and to identify alternative insecticides.
